# Arch and Frozen Elephant Trunk Repair for Aortic Dissection in Pregnancy With Situs Inversus Totalis

**DOI:** 10.1093/icvts/ivag223

**Published:** 2026-08-17

**Authors:** Yi Yang, Yipeng Ge, Chengnan Li, Junming Zhu

**Affiliations:** Department of Cardiovascular Surgery, Beijing Aortic Disease Center, Beijing Anzhen Hospital, Beijing Institute of Heart, Lung and Blood Vessel Diseases, Capital Medical University, Beijing 100029, China; Department of Cardiovascular Surgery, Beijing Aortic Disease Center, Beijing Anzhen Hospital, Beijing Institute of Heart, Lung and Blood Vessel Diseases, Capital Medical University, Beijing 100029, China; Department of Cardiovascular Surgery, Beijing Aortic Disease Center, Beijing Anzhen Hospital, Beijing Institute of Heart, Lung and Blood Vessel Diseases, Capital Medical University, Beijing 100029, China; Department of Cardiovascular Surgery, Beijing Aortic Disease Center, Beijing Anzhen Hospital, Beijing Institute of Heart, Lung and Blood Vessel Diseases, Capital Medical University, Beijing 100029, China

**Keywords:** aortic dissection, pregnancy, situs inversus, dextrocardia, aortic aneurysm, frozen elephant trunk

## Abstract

**Objective:**

Acute type A aortic dissection during pregnancy with situs inversus totalis is exceptionally rare.

**Methods:**

We report a 27-year-old woman at 24 weeks of gestation who underwent emergency ascending aortic and total arch replacement with frozen elephant trunk implantation.

**Results:**

Fetal demise occurred on postoperative day 1, but the mother recovered without neurological deficit and remained well at 10-year follow-up.

**Conclusions:**

This case demonstrates the feasibility of complex arch repair in mirror-image anatomy and highlights the importance of individualized operative planning and multidisciplinary maternal-fetal management in experienced aortic centres.

## INTRODUCTION

Acute type A aortic dissection is rapidly fatal, whereas pregnancy creates competing maternal and foetal priorities.^1^^,^^2^ Before foetal maturity, maternal rescue is generally prioritized. Situs inversus totalis reverses cardiac, caval and arch anatomy, altering cannulation, cerebral perfusion and graft orientation.^3^ Extensive arch repair during pregnancy with situs inversus totalis has not been reported.

## CASE REPORT

**Figure 1.**
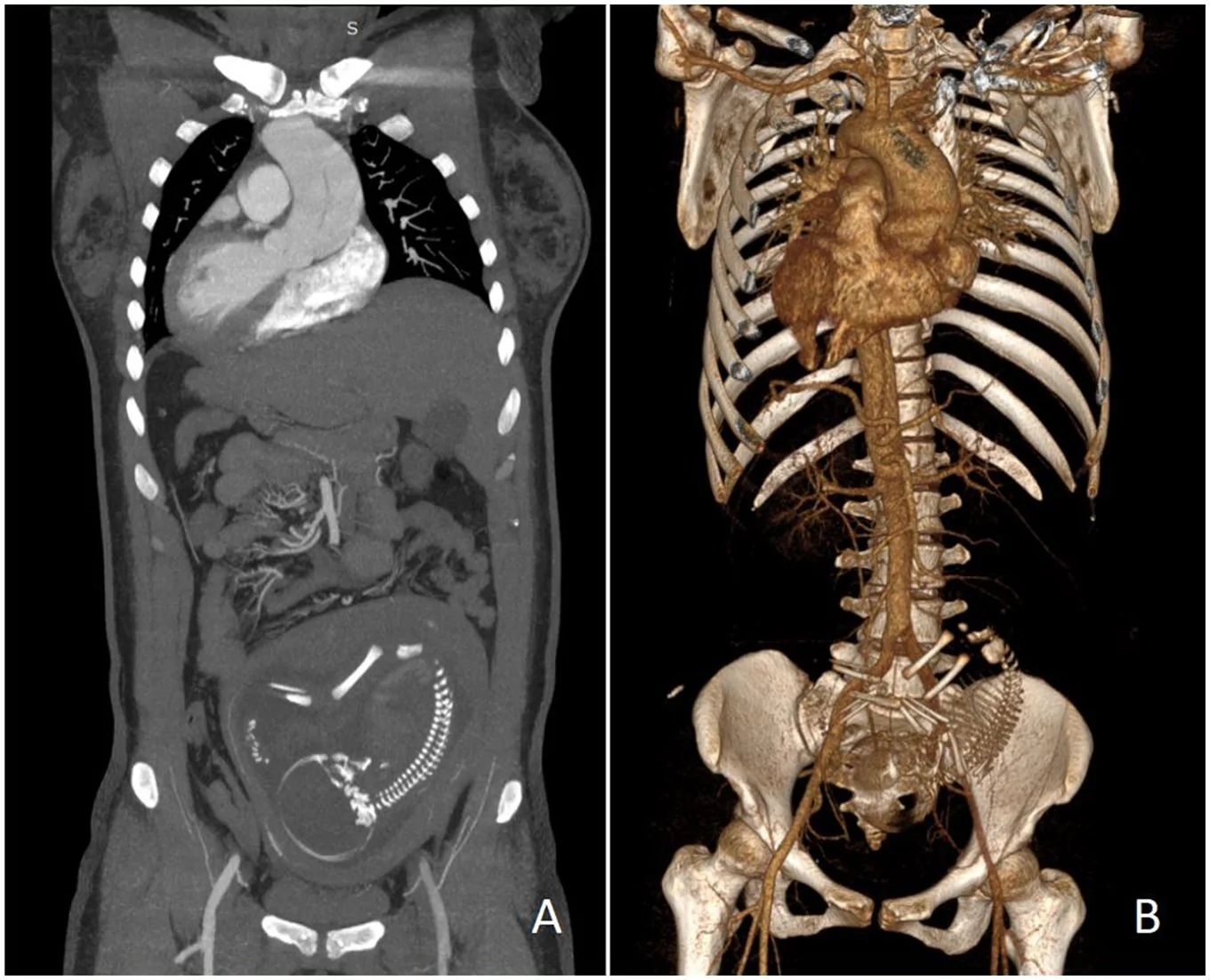
Preoperative Computed Tomography Angiography (CTA). (A) Coronal view showing situs inversus totalis with dextrocardia and a gravid uterus. (B) Three-dimensional reconstruction showing mirror-image anatomy and a reversed aortic arch.

**Figure 2.**
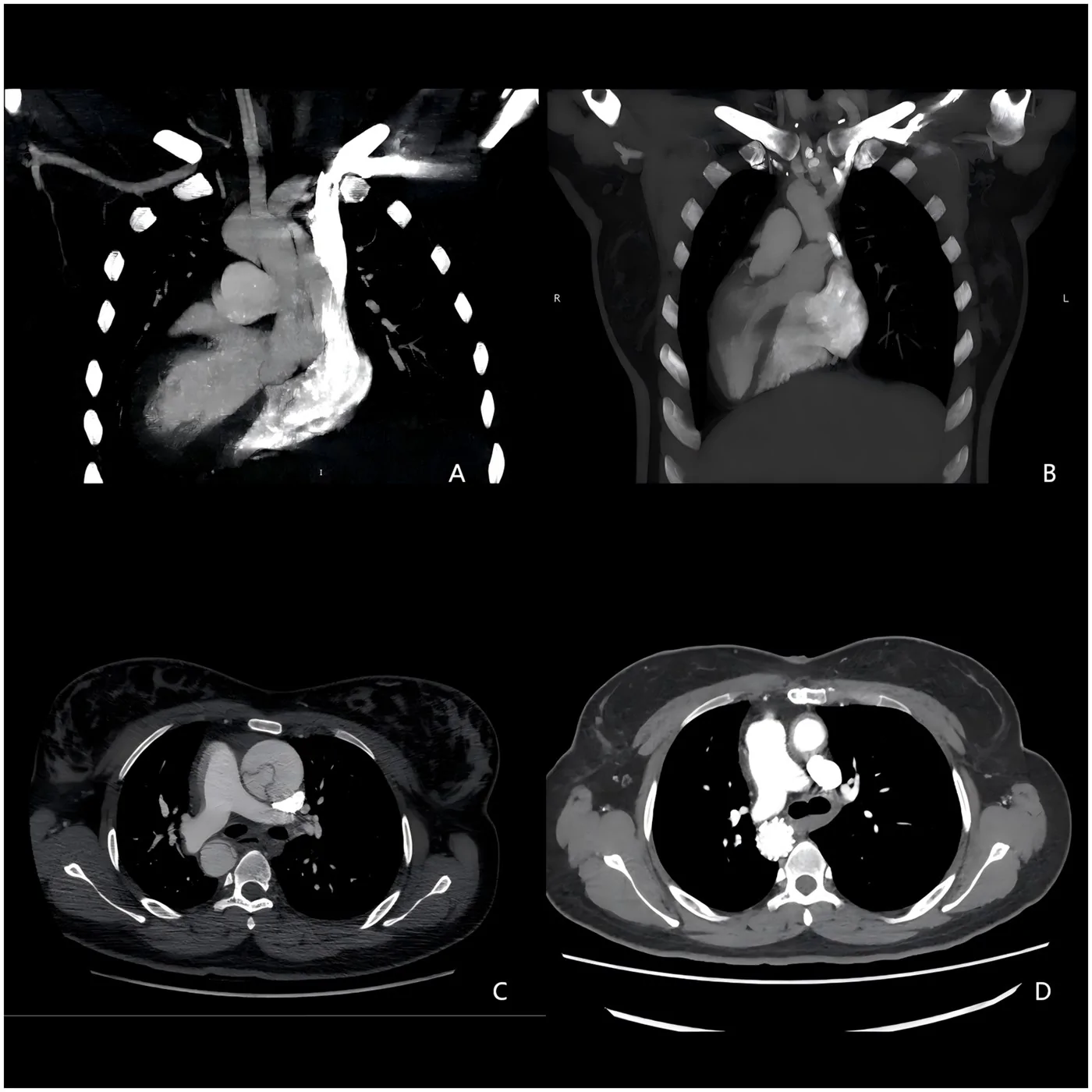
Preoperative and Postoperative Computed Tomography Angiography (CTA). (A) Preoperative coronal image showing supra-aortic dissection and nonopacification of the distal left brachiocephalic artery. (B) Postoperative coronal image showing patent reconstructed supra-aortic branches. (C) Preoperative axial image showing an ascending aortic intimal flap. (D) Ten-year follow-up axial image showing thrombosis of the false lumen around the stent-graft.

A 27-year-old woman at 24 weeks of gestation presented with chest and back pain for 12 h. She had a 4-year history of hypertension and pregnancy-associated hypertension but had stopped antihypertensive therapy. Blood pressure was 200/120 mmHg. No clinical features suggested connective-tissue disease. Echocardiography showed an ascending aortic flap, mild regurgitation and a 50-mm ascending aorta. Contrast-enhanced computed tomography angiography confirmed acute type A dissection, situs inversus totalis and a viable foetus (**Figures 1 and 2C**).

After multidisciplinary discussion, emergency maternal rescue surgery was undertaken with continuous foetal monitoring. Sternotomy confirmed mirror-image anatomy. Preoperative CTA showed dissection of all 3 supra-aortic origins and nonopacification of the distal left brachiocephalic artery (**Figure 2A**), precluding reliable left axillary cannulation. Cardiopulmonary bypass used the right femoral artery and left-sided morphologic right atrium, with direct true-lumen left common carotid perfusion.

The ascending aortic tear extended into the arch and descending aorta. A 22-mm 4-branched graft was anastomosed at the sinotubular junction. At 23.6 °C, a 6-mm cannula was placed in the true lumen of the left common carotid artery for cerebral perfusion at 5 mL/kg/min with cerebral oximetry. A 26 × 100-mm frozen elephant trunk was deployed in the descending true lumen. Lower-body perfusion was restored and the arch branches reconstructed. Cardiopulmonary bypass, cross-clamp, and cerebral perfusion times were 223, 105, and 22 min, respectively.

Twelve units of red cells and 400 mL of plasma were transfused. Foetal demise was detected on postoperative day 1. Maternal hypoxaemia later required reintubation; caesarean delivery was performed with uterine preservation because the retained demised foetus was considered to add to the respiratory burden. The mother recovered without neurological deficit and was discharged after 15 days. At 10-year follow-up, she remained well, had no subsequent pregnancy, and CTA showed patent reconstructed supra-aortic branches, residual descending dissection and thrombosis of the false lumen around the stent graft (**Figure 2B and D**).

## DISCUSSION

Pregnancy-associated acute type A dissection threatens mother and foetus, while situs inversus totalis creates a mirror-image operative field. Previous reports of type A dissection in situs inversus totalis^3^ did not address pregnancy with extensive arch-vessel involvement. Operative planning must therefore integrate gestational timing with the anatomic consequences of mirror-image circulation.

In our 25-year experience of 60 patients with pregnancy- or postpartum-related type A dissection, foetal mortality was 11.1% with delivery first, 22.6% with simultaneous delivery and repair, and 66.7% when aortic repair was performed first.^2^ Before foetal maturity, maternal rescue may be necessary despite uncertain foetal survival. During CPB, adequate flow, perfusion pressure, oxygen delivery, and haematocrit are important for uteroplacental perfusion; unnecessary hypothermia and prolonged circulatory arrest should be avoided.

Foetal demise was probably multifactorial, with potential contributions from the dissection, prolonged CPB, circulatory arrest at 23.6 °C, haemodynamic fluctuations, altered uteroplacental perfusion, and transfusion. Because foetal demise preceded later maternal hypoxaemia and reintubation, hypoxaemia should not be considered its primary cause. Careful respiratory management is warranted after prolonged CPB and hypothermia. This was an early case in our experience, and our multidisciplinary strategy has since evolved.

The operative strategy reflected patient-specific anatomy. Dissection of all 3 supra-aortic origins and near occlusion of the distal left brachiocephalic artery precluded reliable left axillary cerebral protection. Femoral inflow and direct true-lumen left common carotid perfusion were therefore used. Total arch replacement reconstructed the cerebral vessels, while frozen elephant trunk implantation expanded the descending true lumen and reduced distal malperfusion that could compromise maternal systemic and uteroplacental perfusion. This standardized strategy was supported by institutional experience^4^ and pregnancy-associated aortic surgical experience.^5^ It should not be routine in pregnancy; repair extent and perfusion should be individualized according to gestational age, branch-vessel involvement, distal pathology, and institutional expertise.

## CONCLUSION

For extensive acute type A dissection in pregnancy with situs inversus totalis, total arch replacement with frozen elephant trunk may be feasible in experienced centres when the arch vessels and descending aorta are involved. Maternal technical success does not ensure foetal safety. Individualized perfusion, minimized CPB and circulatory-arrest exposure, and appropriate counselling remain essential.

## Data Availability

The data underlying this article are available within the article; further de-identified information is available from the corresponding author on reasonable request.
